# Influence of occlusal loading on peri-implant inflammatory cytokines in crevicular fluid: a prospective longitudinal study

**DOI:** 10.1186/s40729-020-00262-2

**Published:** 2020-10-28

**Authors:** Jose Viña-Almunia, Hilario Pellicer-Chover, Berta García-Mira, Javier Romero-Millán, David Peñarrocha-Oltra, Miguel Peñarrocha-Diago

**Affiliations:** Oral Surgery and Implantology Unit, Department of Stomatology, Faculty of Medicine and Dentistry, University of Valencia, Gascó Oliag 1, 46010 Valencia, Spain

**Keywords:** Occlusal loading, Crevicular fluid, Peri-implant clinical parameters, Cytokines

## Abstract

**Objective:**

The objective of the study was to evaluate the relation between occlusal loading and peri-implant crevicular fluid cytokine expression in patients with implant-supported complete fixed prostheses in both arches.

**Material and methods:**

A prospective longitudinal clinical study was performed at a university clinic. Fifteen patients were selected and 11 were included. All patients had bimaxillary implant-supported complete fixed ceramo-metallic prostheses loaded at least 12 months before the beginning of the study. Allocation was established for each patient using a computerized occlusal analysis system. The test implant was the maxillary implant closest to the point of highest occlusal loading. The maxillary implant with least loading was the control implant. Occlusal adjustment was performed using a round diamond burr. This occlusal distribution was verified with the occlusal analysis system. Expression of cytokines from peri-implant crevicular fluid (TNF-α, IL-10, IL-6, IL-1β, IL-8) were recorded and analyzed in both test and control implants before (baseline: T0) and 2 (T1) and 12 months (T2) after occlusal adjustment. The Brunner-Langer non-parametric test was performed.

**Results:**

At T0, the expression of IL-10 was significantly higher in the test group implants (*p* = 0.018). Between T0 and T1, the expression of all the cytokines decreased in the implants of both groups with statistically significant differences, except for TNF (*p* = 0.271). When comparing both groups at T1, there was no statistically significant difference in any of the analyzed cytokines. At T2, TNF-α suffered when compared with baseline, a statistical decrease in both study and control implants (*p* < 0,001). At T2, there were no statistically significant differences between groups in any of the cytokines analyzed.

**Conclusions:**

Implants with higher occlusal load presented higher expression of IL-10 in peri-implant crevicular fluid. Occlusal adjustment produced a decrease in the expression of all the analyzed cytokines, both in test and control implants.

## Introduction

Occlusal forces affect the peri-implant bone. Mechanical stress can have both positive and negative consequences for bone tissue [1] and consequently in osseointegration [2]. Peri-implant crevicular fluid (PICF) might reflect the local peri-implant heath status [3]. Patients presenting peri-implantitis usually show an increased PICF volume [4]. In fact, levels of inflammatory mediators in PICF have been proposed as a measure of active peri-implantitis. Since this disease might be latent in its early stages, biomarker analysis in PICF might serve as a tool for early diagnosis and/or determination of patient susceptibility [3, 5].

With animal experimental studies showing conflicting results, it is unclear whether occlusal overload might cause peri-implant marginal bone loss or total loss of osseointegration [6]. While some studies [7] have shown loss of osseointegration due to overload, others [8, 9] have shown, if there is absence of peri-implant mucosa inflammation, no differences or even higher bone to implant contact. In fact, different literature reviews [10, 11] agree that overload cannot lead to peri-implant bone loss, except in case of bacterial inflammation. Nevertheless, the intensity of the overload might have effect on osseointegration, as demonstrated by the study performed by Miyata et al. [12].

In a previous human study [13], our group found that implants receiving higher occlusal loading presented significantly higher PICF volumes than implants with less occlusal loading. Two and 12 months after occlusal adjustment, PICF volumes were similar in both groups. There were no statistical differences between both groups in the other clinical parameters measured, i.e., probing depth, bleeding on probing, recession of the mucosal margin, and keratinized mucosa width. This article is a second part of this previous study.

So, the aim of this prospective longitudinal study was to evaluate the relation between occlusal loading and peri-implant crevicular fluid cytokine expression in patients with implant-supported complete fixed prostheses in both arches.

## Materials and methods

### Ethical statement

The study fulfilled Declaration of Helsinki principles for medical research involving humans. All patients gave their informed consent to take part, and the study was approved by the University of Valencia Ethics Committee (ref no. H1335344280712).

### Patient selection and study design

This article is the second part of a prospective clinical study that took place at the Oral Surgery Unit at Valencia University [13] with a 12-month follow-up. Fifteen patients, who had been rehabilitated with ceramo-metallic complete fixed prostheses, supported by 8 Phibo TSA® implants (Phibo Dental Solutions®, Impladent, Senmenat, Barcelona, Spain) in the upper maxillary and 6 in the mandible were selected. Cemented ceramo-metallic prostheses were drilled in chromium-cobalt at the facility of the manufacturer (Phibo Dental Solutions®, Impladent, Senmenat, Barcelona, Spain) and subsequently coated with feldspathic ceramic. Premier Implant (Plymouth meeting, PA, USA) was used to cement the prostheses.

Patients had to fulfill the following inclusion criteria: adult patients (> 18 years) rehabilitated with fixed full-arch prosthesis loaded at least 12 months before the study and signature of informed consent document. The exclusion criteria were as follows: use of local or system antimicrobials 3 months prior to the study, smokers, pregnant or lactating women, patients with a history of bisphosphonate therapy, patients who had malignant diseases or other diseases treated with chemotherapeutic agents (“chemotherapy”) or head and neck radiotherapy during the past 5 years, severe bruxism, poor oral hygiene, incomplete data gathering, or failure to attend scheduled control visits.

**Fig. 1:**
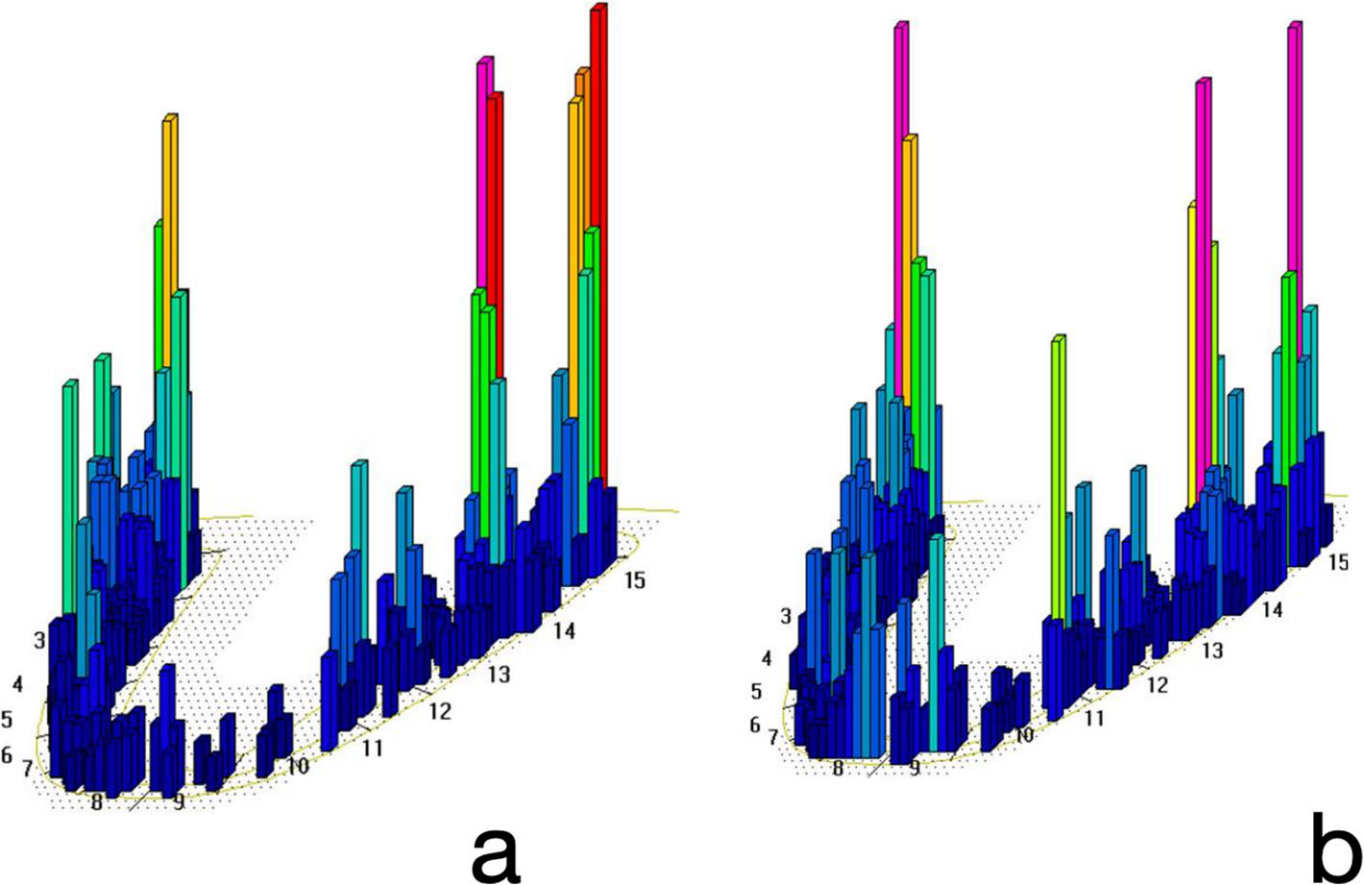
Occlusal analysis with the T-scan®III system. **a** Before adjustment (T0), when the study groups were stablished, 26 were considered the study implant and 22 the control implant. **b** After occlusal adjustment. Note that before occlusal adjustment, there are red bars at the left side (study implant), while after adjustment, the red bars disappear and the occlusion is distributed

After implant loading, the occlusal adjustment was checked with 12 μm articulating paper; then, all the patients had a follow-up at 1 week; 1, 3, and 12 months; and once per year in order to control the occlusal distribution. Patients received rigorous professional prophylaxis with Teflon curettes and rotary instrument brushing, and instructions were given for improving and maintaining oral hygiene at home. Patients then underwent an occlusal analysis with a computerized system (T-scan®III, Tesco, South Boston, USA), where the patient makes mastication movements that activate a sensor placed between the dental arches, while the computer registers and processes the data. Occlusal contacts are represented on screen by topographic images that describe the shape of the contact areas, the relative force, the surface area, and the time sequence of occlusal contacts. Differences in occlusal loading are shown as color changes, ranging from red (high loads), graduating through the colors of the spectrum, to blue (low loads). It is important to underline that before the start of the study, patients’ comfort and chewing were correct. Occlusal adjustment did not produce changes in the chewing perception of the patient. Implants presented healthy peri-implant conditions as an inclusion criterion, so changes in cytokine expression would be due to the effect of occlusal loading. Through the occlusal analysis system, two implants per patient were established:

- Test implant—maxillary implant closest to the point of highest occlusal loading.
- Control implant—maxillary implant closest to the point of least loading. In the case of finding 2 implants with least loading, the furthest from the study implant was selected as the control implant (Fig. 1a).

### Occlusal adjustment and data collection

**Fig. 2:**
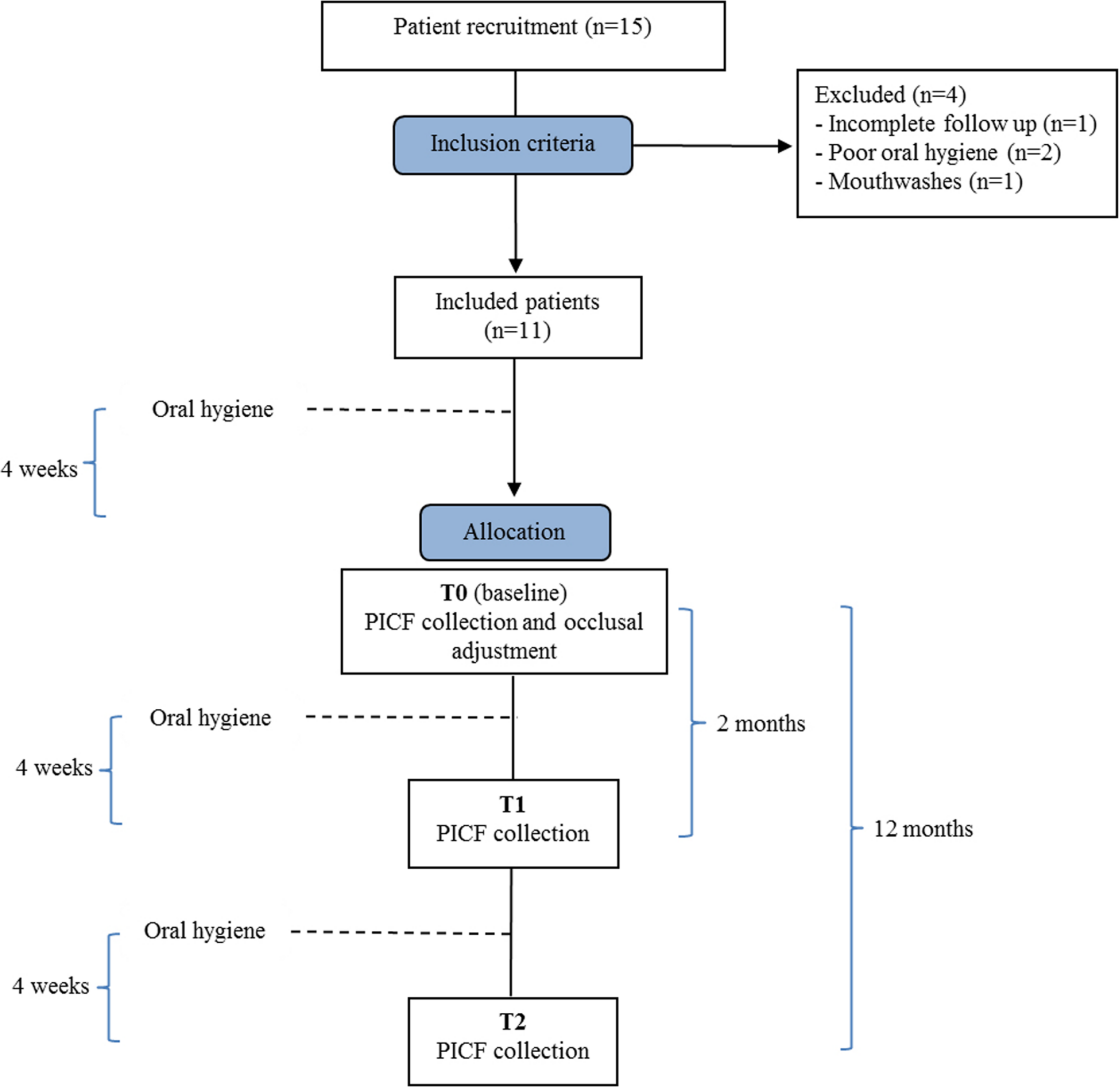
Study timeline. An oral hygiene was performed 4 weeks every each PICF collection. PICF collection was carried out, in study and control implants, before occlusal adjustment (T0) and 2 (T1) and 12 months after occlusal adjustment (T2)

One month after oral hygiene, at baseline, PICF from test and control implants was collected (T0), and occlusal adjustment was performed to distribute occlusal loading evenly over the whole arch. Occlusal distribution was verified with the T-Scan®III (Fig. 1b), following the method described by Kerstein [14]. PICF was again collected 2 (T1) and 12 months (T2) after occlusal adjustment. In T1 and T2, occlusal distribution was checked but no modification was done. Only at T2 and after registering all the studied parameters and taking the PICF collection, occlusal adjustment was done if necessary. Figure 2 outlines the timeline of the study, including the occlusion adjustment and the PICF collection periods.

The following cytokine expression (TNF-α, IL-10, IL-6, IL-1β, IL-8) was measured from the PICF. Cytokine expression between test and control implants was compared in each timepoint and between timepoints. Rigorous professional prophylaxis, as described above, was performed 4 weeks before every PICF collection.

### PICF volume collection and cytokine analysis

PICF was collected from the implants selected for study by inserting sterile paper strips (Periopaper Strip® Proflow Incorporated, New York, NY, USA). The technique consisted of air-drying the mouth, isolating the area with cotton wool rolls, and gentle drying of the implant area where the paper strip was to be placed. PICF sample collection was performed by inserting the Periopaper Strip® in the peri-implant sulcus for 30 s.

Each sample was diluted in an Eppendorf tube (Millipore, Massachusetts, USA), with 200 mL of 50 mM phosphate buffer, pH 7.2, together with a pool of protease inhibitors (Roche Diagnostics GmbH, Mannheim, Germany) and 0.1 mM phenyl sulfony l fluorate, and incubated for 2 h. The samples were centrifuged at 1000*g* for 5 min, and the supernatant was stored at − 80 °C until use.

TNF-α, IL-10, IL-6, IL-1β, and IL-8 were evaluated in the supernatants stored at − 80 °C. The evaluation was performed using the Human Inflammation Cytometric Bead Array system (Becton Dickinson, BD Biosciences, San Diego, CA, USA) and cytofluorometry analysis (Becton Dickinson, BD Biosciences, San Diego, CA, USA). The samples and positive controls (standard curve) were processed according to the instructions of the manufacturer, and the values for TNF-α, IL-10, IL-6, IL-1β, and IL-8 were calculated and reported as picograms per milliliter. Data were acquired with a fluorescence-activated cell sorter Microbiology Calibur flowcytometer (Becton Dickinson, Franklin Lakes, NJ, USA).

### Statistical analysis

The statistical analysis used SPSS statistical software for Windows (version 15.0, SPSS Inc., Chicago, IL, USA). The Brunner-Langer non-parametric test was performed. Statistical significance was established as *p* < 0.05.

## Results

### Study population

Fifteen patients were preliminarily enrolled in the study. Four of them did not fulfill the inclusion criteria: one for failing to attend scheduled appointment, 2 for plaque scores > 25%, and one for using mouthwashes. This left 11 included patients, 4 women and 7 men, with a mean age of 58.4 years. Figure 2 shows the study timeline and patient enrollment.

### Peri-implant cytokine analysis

Before occlusal adjustment (T0; baseline), test group implants expressed higher concentration of IL-6, TNF-α, and IL-10, but significant differences were only found in IL-10 (*p* = 0.018).

Two months after occlusal adjustment (T1), all the cytokines analyzed decreased when compared with baseline (statistically significantly), in both test and control implants except for TNF-α that decreased without significant differences (*p* = 0.271). At this timepoint (T1), IL-10, IL-6, and IL-1β were highly expressed in study implants but there were no statistically significant differences in none of the cytokines between test and control implants.

**Fig. 3:**
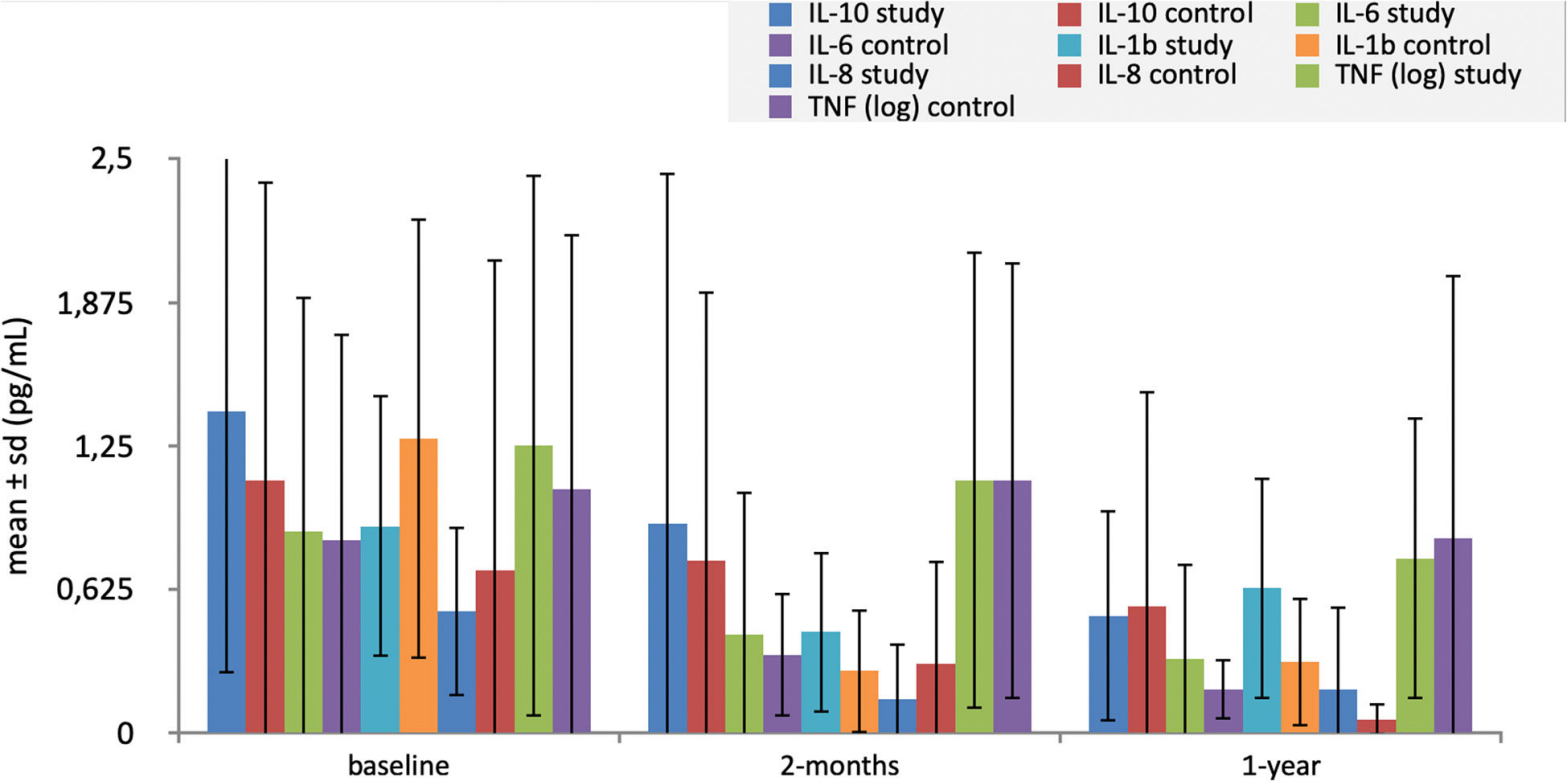
Cytokine expression in PICF from baseline (T0) and 2 (T1) and 12 months after adjustment (T2). At baseline, the expression of IL-10 was significantly higher in the test group implants. Between baseline and 2 months, all the cytokines decreased in the implants of both groups with statistically significant differences, except for TNF. When comparing both groups 2 months after occlusal adjustment, there was no statistically significant difference in any of the analyzed cytokines. One year after occlusal adjustment, TNF-α suffered when compared with baseline, a statistical decrease in both study and control implants. At T2, there were also no statistically significant differences between groups in any of the cytokines. TNF is expressed with a logarithm in order to use it in the same graphic than the other cytokines

Twelve months after adjustment (T2), all the cytokines including TNF-α significantly decreased when compared with baseline, in both test and control implants (*p* < 0,001). At this timepoint (T2), IL-6, IL-1β, and IL-8 showed higher values in study implants, but there were no significant differences between groups in all the cytokines analyzed. TNF-α showed a tendency to significance with higher values in the test group (*p* = 0.085). Figure 3 summarizes the results of the cytokine expression in PICF and the evolution of these proteins during the study period.

## Discussion

The aim of this study was to evaluate the relation between occlusal loading and PICF cytokine expression in patients with implant-supported complete fixed prostheses in both arches. The results show that implants with higher occlusal load presented higher IL-10 levels than control implants. It was also observed that occlusal adjustment diminished all the cytokines analyzed, in a short and medium period of time, in both test and control implants. Specifically, 2 months after occlusal adjustment, all the cytokines decreased significantly in both study and control implants, except TNF-α that decreased significantly at 12 months.

IL-10 is a cytokine with potent anti-inflammatory properties [15–17] that regulates the production of proinflammatory cytokines such as IL-1β, IL-6, IL-8, or TNF-α [18–21]. At baseline, we found that IL-10 was significantly increased in the test implant. In other words, IL-10 is expressed to counteract the expression of proinflammatory cytokines. This may be a consequence of local immune response against the proinflammatory cytokines released as a consequence of higher occlusion loading. Zani et al. [22] pointed that combination of biomarkers increased considerably the diagnostic properties compared to isolated biomarkers. That is the reason why we included proinflamatory (IL-1β, IL-8, or TNF-α) and anti-inflammatory cytokines (IL-10). There are no studies that analyze the effect of occlusion on PICF cytokine expression, but biomarkers and enzymes in PICF have shown promising results in differentiating from peri-implant disease to healthy [22–25]. Liskmann et al. [23] found significant higher values of IL-6 in the saliva of peri-implantitis patients compared with healthy patients. They also found that IL-10 was present in patients with peri-implantitis while in healthy patients it was not detectable. Severino et al. [25] found significant higher PICF IL-6 expression in patients with peri-implantitis compared with healthy patients, but no significant differences were found in IL-10 between groups. So studies evaluating the relation between health and disease implant tissues and cytokine expression showed indicate moderate evidence and sometimes contradictory results. The most used biomarkers to assist the early diagnosis of peri-implantitis are IL-1β and TNF-α [26]. Studies showed that higher levels of IL-1β [27] and TNF-α [28] both in gingival crevicular fluid and PICF are associated with periodontitis and peri-implantitis, although there are contradictory results [29]. In the present study, there were no significant differences in these proinflammatory cytokines between groups. It is important to underline that implant rehabilitation was performed properly and there was no discomfort during chewing in any patient before the patients were included in the study. Through the occlusal analysis system, it was shown that there was an implant with higher occlusal load. It was not overloaded since as we published before the clinical parameters were correct and the patients did not feel discomfort during chewing. So the occlusal adjustment was performed at a subclinical level (the patient did not feel any difference after the adjustment). The analysis of the changes in the cytokine expression is a subclinical parameter and is a complementary analysis to the changes in the PICF volume that we published before [13]. This subclinical level (there is no pathology) might be the reason why, before occlusal adjustment, some cytokines are highly expressed in the test implants while some others presented higher values in the control implants. An interesting fact is that after occlusal adjustment, all the cytokines analyzed decreased not only in the test but also in the control group implants. This effect could be due a stabilization of the cytokine expression in all the implants caused by the splinting of the implants. TNF-α induces fibroblast apoptosis and reduction of the repair capacity of the peri-implant tissue, but mechanical therapy seems to revert this situation [30]. IL-1β regulates the degradation of extracellular matrix components of the plasminogen system and the collagenase activity in inflammation and wound healing [31]. These differences between cytokine features might explain why TNF-α decreased later. Using the same sample in a previous study [13], we showed that PICF volume was statistically higher in test implants and that after occlusal adjustment, a significant decrease was observed. It was also observed that clinical parameters were similar between groups and did not change after occlusal adjustment. It is important to underline that PICF and the cytokines presented on it are a subclinical parameter, but this is having great interest in the scientific community in order to detect peri-implant diseases in early stages. Tözüm et al. [26] pointed in a recent consensus review that prospective longitudinal studies with periodical PICF collection and with appropriate number of implants are needed. Due to a cyclic progression of peri-implant diseases, cross-sectional studies with a single moment of fluid collection are not well designed. The present investigation is a prospective longitudinal study where samples were collected 3 times from baseline to 1 year follow-up after occlusal adjustment.

The data that the scientific literature provides about the influence of occlusal loading on peri-implant tissue health is basically from animal studies. The reason is that it is unethical to do an implant rehabilitation with supraoclusal contacts deliberately [10, 11]. The present study provides information of cytokines present in PICF of implants with different occlusal loading degrees. The results showed that peri-implant tissue health, at least in a subclinical level (cytokines in PICF), is related to occlusal loading. It is important to underline that at baseline, the included patients did not feel any discomfort at chewing or at occlusion. Articulation paper (12 μm thick) was used to do the final adjustment in the occlusion when the implants were loaded. However, it was possible to discern between implants close and far away from the highest point of occlusal load thanks to the T-scan®III occlusal analysis system, a non-invasive method.

Overload is defined as the occlusal forces that exceed the mechanical or biological load-bearing capacity of the osseointegrated oral implants or the prosthesis, causing either a mechanical failure or a failure in the osseointegration [2]. However, there is an inappropriate use of the term overload, since it should lead to a catabolic reaction of the bone [1], but different studies [9, 32] showed an anabolic rather than a catabolic effect of “overload,” in bacterial unchallenged peri-implant bone tissues. In the present study, the term “point of highest occlusal loading” was used as the patients were rehabilitated not producing a hypercontact, and this point of higher occlusal load was determined only with the help of the T-scan®III. Studies performed in animals have shown different results. While some studies observed an increased BIC (bone implant contact) in overloaded implants [8, 9], others observed loss of osseointegration [7]. An explanation for the difference in outcome of these studies may be related to the animals used, i.e., dogs and monkeys; the higher bone density of the bone surrounding the implant in the study by Heitz-Mayfield et al. [8]; and the lateral direction of the load used by Isidor [7].

Results from the present investigation should be interpreted with caution due to the limitations of the present study. The sample met the strict inclusion criteria such as the homogeneity in the number of implants per patient, the type of rehabilitation, and the oral hygiene of the patients, but studies with larger number of patients are necessary. In order to reduce bias, the present study was performed with patients that had a very good oral hygiene with full-mouth plaque and full-mouth bleeding scores < 25%. It would be interesting to analyze the effect of occlusion and clinical and subclinical parameters (cytokines in PICF) in patients with a peri-implant disease established. These would help to understand, together with clinical and radiological parameters, the influence of loading in the peri-implant tissues.

The present study concludes that implants with higher occlusal load presented higher levels of IL-10 in peri-implant crevicular fluid. Occlusal adjustment produced a decrease in the expression of all the cytokines analyzed, both in test and control implants.

## Data Availability

The datasets used or analyzed during the current study are available from the corresponding author on reasonable request.

## Abbreviations

T0: Baseline
T1: Two months follow-up
T2: Twelve months follow-up
PICF: Peri-implant crevicular fluid
BIC: Bone implant contact
